# Effect of host‐protein test (TRAIL/IP‐10/CRP) on antibiotic prescription and emergency department or urgent care center return visits: The JUNO pilot randomized controlled trial

**DOI:** 10.1111/acem.70031

**Published:** 2025-04-18

**Authors:** Adam J. Singer, Judd E. Hollander, Efrat R. Kean, Hope Ring, W. Frank Peacock, Karina M. Soto‐Ruiz, Sergey Motov, Joby Thoppil, Phyllis Hendry, Salim Halabi, Andrew C. Meltzer, Gary F. Headden, Tal Brosh‐Nissimov, David Zeltser, Chad M. Cannon

**Affiliations:** ^1^ Renaissance School of Medicine at Stony Brook University Stony Brook New York USA; ^2^ Sidney Kimmel Medical College of Thomas Jefferson University Philadelphia Pennsylvania USA; ^3^ Emergency Medicine University of Kansas Medical Center Kansas City Kansas USA; ^4^ Emergency Medicine Baylor College of Medicine Houston Texas USA; ^5^ Emergency Medicine Comprehensive Research Associates, LLC Houston Texas USA; ^6^ Emergency Medicine Maimonides Medical Center Brooklyn New York USA; ^7^ Emergency Medicine UT Southwestern Medical Center Dallas Texas USA; ^8^ Emergency Medicine University of Florida College of Medicine Jacksonville Florida USA; ^9^ Emergency Medicine Carmel Medical Center Haifa Israel; ^10^ Emergency Medicine George Washington University School of Medicine and Health Sciences Washington DC USA; ^11^ Emergency Medicine The Medical University of South Carolina Charleston South Carolina USA; ^12^ Emergency Medicine Assuta Ashdod Medical Center Ashdod Israel; ^13^ Emergency Medicine Tel Aviv Sourasky Medical Center ‐ Ichilov Tel‐Aviv Israel

**Keywords:** antibiotics, bacterial, diagnostics, rapid host response test, viral

## Abstract

**Objectives:**

Determining etiology for adults with symptoms of lower respiratory tract infection (LRTI) is challenging. MeMed BV (MMBV), an FDA‐cleared blood test, computationally integrates the levels of three host proteins to differentiate bacterial and viral infections. We evaluated MMBV's impact on safe antibiotic prescribing at emergency department/urgent care centers (ED/UC).

**Methods:**

The JUNO randomized controlled trial (RCT; NCT05762302) was a prespecified pilot phase of the JUPITER RCT. JUNO enrolled adult ED/UC patients with LRTI symptoms and clinician's consideration for antibiotic treatment. Inclusion criteria were fever within 7 days and one of cough, sputum production, dyspnea, or auscultation abnormality. Exclusion criteria were prior antibiotic use or immunosuppression. Patients were randomized to standard care (SC) or SC plus MMBV arms. JUNO's primary objective was to assess antibiotic prescription rate in the SC arm; the secondary objective was to assess JUPITER's study design.

**Results:**

Eleven centers randomized 260 patients, with 214 included (106 SC, 108 MMBV). Median (IQR) age was 40 (28–55.8) years, 57% were female, and 78.5% were enrolled at ED. Common symptoms were cough (93.0%) and chills (70.0%). Overall, antibiotic prescription rates were 30% (95% CI 22% to 40%) and 24% (95% CI 17% to 33%) in the SC arm versus the MMBV (absolute difference of −6% [95% CI −18% to 6%]). More antibiotics were given with bacterial MMBV scores (41% vs. 78%, absolute difference 37%, 95% CI 6% to 61%; *n* = 40) and less with viral MMBV scores (25% vs. 12%, absolute difference –13%, 95% CI −25% to 0%; *n* = 144) in the SC versus MMBV arms. There was no increase in ED/UC return visits (8% vs. 3%, difference –6%, 95% CI −12% to 1%) or hospitalizations (3% vs. 0%, difference –3%, 95% CI −7% to 1%) in the SC arm versus the MMBV arm.

**Conclusions:**

JUNO demonstrated that JUPITER's design results in 30% antibiotic prescription rate in the SC arm. JUNO supports that MMBV optimizes antibiotic prescriptions without increasing return ED/UC visits or hospitalizations.

## INTRODUCTION

Determining infection etiology in patients presenting with symptoms of lower respiratory tract infection (LRTI) is challenging as bacterial and viral infections often present similarly.^1^ This diagnostic uncertainty confounds antibiotic prescription decisions. Unnecessary antibiotic use not only contributes to emerging antibiotic resistance but also exposes patients to unnecessary side effects and costs. On the other hand, delayed or no antibiotic treatment to patients with bacterial infection can lead to poor patient outcomes and return visits,^1^, ^2^, ^3^ especially for elderly patients and when there is sepsis.^4^

The decision to prescribe antibiotics is commonly guided by laboratory tests and clinical judgment. While biomarkers provide some guidance,^5^, ^6^ their general applicability remains debated.^7^, ^8^ Pathogen detection methods, e.g., cultures and viral panels, may not provide a result during the patient's emergency department/urgent care center (ED/UC) visit. Moreover, when LRTI is suspected, diagnosis is complicated by the difficulty to access pathogens in the lower respiratory tract and the need to discriminate infection from colonization. Furthermore, viral detection does not exclude bacterial coinfection, limiting the utility of pathogen‐based tools.^9^

MeMed BV (MMBV), an FDA‐cleared host‐protein blood test, represents an innovative approach to this problem. By computationally integrating the levels of TNF‐related apoptosis‐induced ligand (TRAIL), interferon gamma–induced protein‐10 (IP‐10), and C‐reactive protein (CRP), MMBV provides a score that differentiates bacterial (or bacterial coinfection) and viral (or other nonbacterial) infections based on predefined score thresholds.^10^, ^11^, ^12^, ^13^, ^14^, ^15^, ^16^, ^17^ MMBV is compatible with serum and whole blood, with a run time of 15 min. Multiple MMBV diagnostic accuracy studies reported high sensitivity and specificity^10^, ^11^, ^12^, ^13^, ^14^, ^15^, ^16^, ^17^, ^18^, ^19^; for example, in adults presenting with symptoms of LRTI (*n* = 415), MMBV attained sensitivity of 98.1% (95% confidence interval [CI] 95.4%–100%) and specificity of 88.4% (95% CI 83.7%–93.1%).^10^ Importantly, MMBV was shown to outperform individual biomarkers, including CRP, WBCs, and procalcitonin and clinician's initial diagnostic suspicion.^10^, ^11^, ^12^, ^13^, ^14^, ^15^, ^16^, ^17^, ^18^, ^19^ Notably, based on these observational studies where MMBV results were not provided to the treating clinician, the impact of MMBV on antibiotic prescription was conjectured by comparing the test result to actual antibiotic use as documented in the medical record.^10^, ^11^, ^14^ Among adults with LRTI, a potential reduction from 56% to 19% was observed that did not cause missed bacterial infections.

Given its high sensitivity and specificity,^10^, ^11^, ^12^, ^13^, ^14^, ^15^, ^16^, ^17^, ^18^, ^19^ real‐world introduction of MMBV is anticipated to “optimize” antibiotic prescription. MMBV achieves not only reduction of unnecessary antibiotics but also reduction of missed bacterial infections. Accordingly, MMBV's absolute impact on antibiotic prescribing rates is expected to be shaped by these two opposing forces: a reduction in antibiotic overuse and an increase in appropriate prescribing to prevent missed bacterial infections. This balance is expected to depend on the patient population and habitual prescribing practices.

To focus on the impact of MMBV specifically on unnecessary antibiotic prescription, we designed the JUPITER randomized controlled trial (RCT). JUPITER recruits patients presenting to the ED/UC with LRTI for whom the clinician has already decided to prescribe antibiotics. These patients are then randomized and only clinicians in the interventional arm are provided with the MMBV result and the opportunity to change their decision. Since JUPITER is the first RCT to assess the impact of MMBV on prescribing, a prespecified pilot phase was conducted to evaluate its design.

Here we describe the results of the JUNO RCT, the prespecified pilot phase of the JUPITER RCT. JUNO's objective was to assess the prescription rate in the standard care (SC) arm and evaluate JUPITER's study design and workflow. Additionally, JUNO assessed JUPITER's outcomes, namely, MMBV's impact on antibiotic prescription and ED/UC return rates. JUNO was also conducted to familiarize the research and clinical staff with MMBV.

## METHODS

### Study design

The JUNO trial was a prespecified pilot phase of the prospective, multicenter, randomized controlled JUPITER trial (NCT05762302). JUNO was intended to determine the prescription rate in the control arm generated by JUPITER's design and workflow. Additionally, JUNO was employed to explore JUPITER's outcomes, namely, MMBV's impact on antibiotic prescription and ED/UC return rates (for more details see Supplementary Methods). The institutional review boards approved the trial (WIRB‐IRB20226229; 0642‐22‐TLV; 0168‐22‐CMC; 0095‐23‐AAA).

### Settings and selection of participants

Adult (≥18 years) ED/UC patients presenting with symptoms of LRTI, for whom clinicians considered antibiotic treatment, were enrolled by convenience sampling during the research team's working hours across 11 sites (working weekdays, 08:00–18:00). By the end of JUNO, all JUPITER sites were enrolling. Patients were eligible if they had experienced fever within the past 7 days and at least one of cough, sputum production, dyspnea, or auscultation abnormality (Table 1). After informed consent was obtained, patients were randomized using 1:1 allocation per a computer‐generated randomization schedule stratified by sex and age group (18 ≤ age < 45, 45 ≤ age < 65, 65 ≤ age). Patients were not stratified according to ethnicity; study population is expected to resemble the U.S. Census Bureau of July 1, 2016. Block sizes of randomization were concealed from clinicians. Patients were randomized into a control or interventional arm. Randomized patients discharged from the ED/UC were eligible for this analysis.

**TABLE 1:** Eligibility criteria.

Inclusion criteria	Exclusion criteria
≥18 years of age	Systemic antibiotics within 72 h prior of enrollment
Current disease duration ≤7 days	Suspicion and/or confirmed diagnosis of infectious gastroenteritis/colitis
Temperature ≥37.8°C (100°F) or tactile fever, noted at least once within the past 7 days	Inflammatory disease requiring treatment (e.g., lupus, other vasculitis)
Clinical suspicion of LRTI and one of the following: cough (new or worsening), sputum production, dyspnea, shortness of breath, chest discomfort, auscultatory abnormality (wheezing, rhonchi)	A proven or suspected infection on presentation with Mycobacterial, parasitic or fungal (e.g., histoplasma) pathogen
Clinician consideration or intent to prescribe antibiotics	Congenital immune deficiency
	HIV, HBV, or HCV infection
	Trauma or surgery requiring hospitalization in the past 7 days
	Pregnancy
	Active malignancy receiving treatment within 6 months
	Treatment with immune‐suppressive/modulating therapies within the past 10 days
	Consider unsuitable for the study by the study team

Abbreviation: LRTI, lower respiratory tract infection.

### Intervention

**FIGURE 1:**
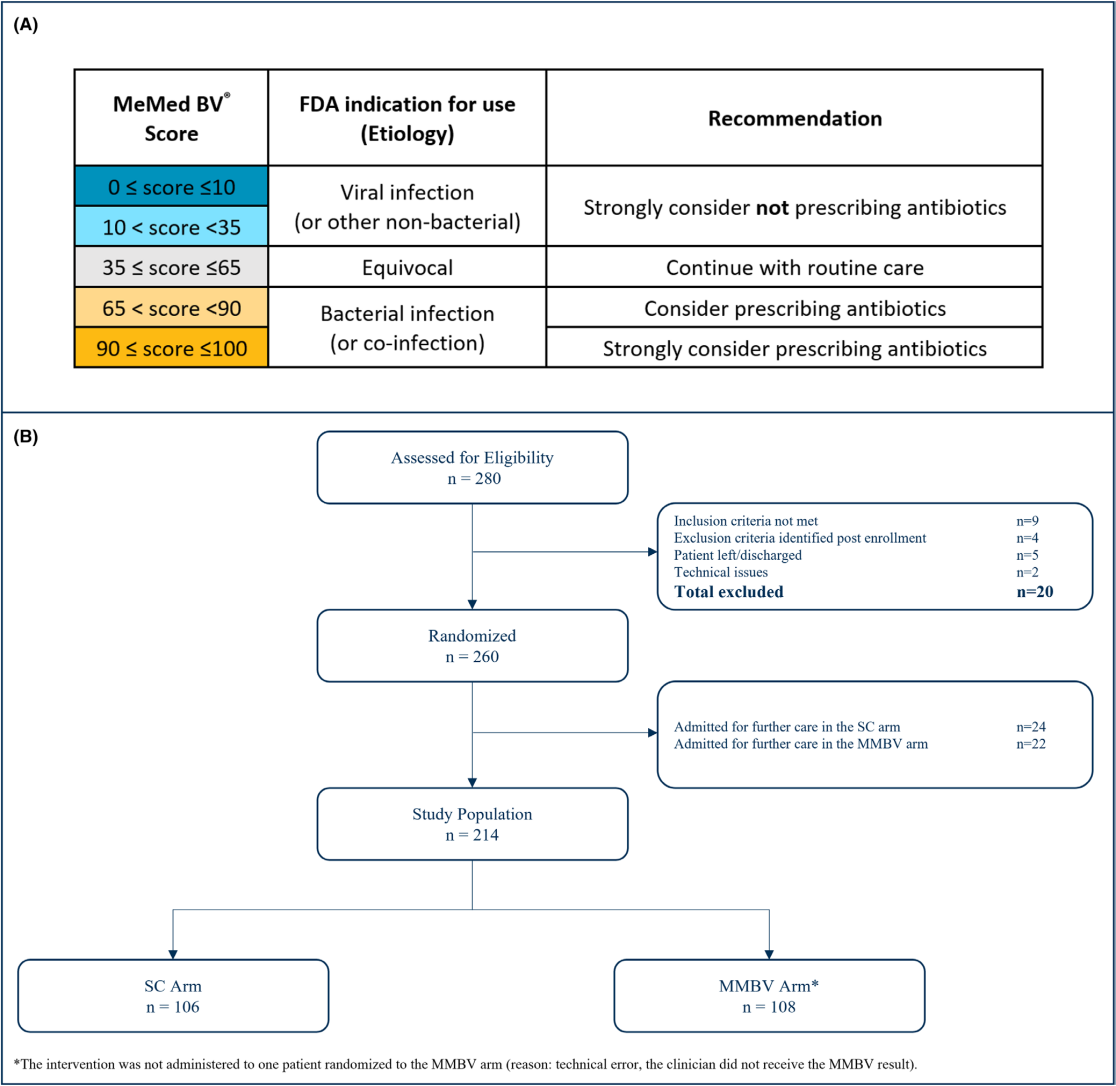
MMBV test results with recommendations and patient flow. (A) MMBV test results, their interpretation and an accompanying recommendation provided to clinicians. This was given to the clinician for patients randomized to the MMBV arm. (B) Patient flow. MMBV, MeMed BV; SC, standard care.

In the control arm patients were treated per SC arm as clinicians in this arm were blinded to MMBV results. In the interventional arm (MMBV arm), clinicians received MMBV results and accompanying practice recommendations (Figure 1A).

### Measurements

For each randomized patient, the MMBV test was run on the MeMed Key (MeMed Inc.) rapid platform,^20^ which provides a score from 0 to 100 based on computational integration of TRAIL, IP‐10, and CRP measurements; the same algorithm as used in previous studies.^10^, ^11^, ^13^, ^15^, ^18^ MMBV tests were executed throughout the research team's working hours (working weekdays, 08:00–18:00). Telephonic follow‐up on Day 28 (±3 days) postrandomization determined whether patients (irrespective of whether recruited at ED or UC) returned to the ED or the UC within 7 days and whether they were hospitalized. For those not answering the call, medical charts were reviewed.

### Outcomes

JUNO's primary outcome was antibiotic prescription rate in the SC arm.

The secondary outcome was to assess JUPITER's study design and to explore JUPITER's outcomes:

1. The primary outcome was antibiotic prescription rate, defined as prescriptions given by the treating clinician in the ED/UC. The primary endpoint was the absolute difference in prescription rate between the arms. We also examined the relative difference in prescription rate, defined as the absolute difference divided by the prescription rate in the SC arm.^21^, ^22^
2. The secondary outcome was the rate of return ED/UC visits within 7 days. The secondary endpoint was the absolute difference in return visit rate between arms. We also examined the rate of ED return visits within 7 days with hospitalization.

JUNO was not powered to detect a statistically significant change in JUPITER's outcomes. The following subgroups were explored:

- All patients eligible for analysis.
- Patients with MMBV scores <35, representing viral infections and accordingly classified as unnecessary antibiotic prescriptions.
- Patients with MMBV scores >65, representing bacterial infections.

To assess the sensitivity of the results to reliance on medical chart review for patients without telephonic follow‐up data, two additional analyses were performed:

- Assuming those without telephonic data returned to the ED/UC.
- Exclusively on those with complete telephonic data.

To assess the sensitivity of the results to inclusion of cases with positive pathogen detection, an additional analysis was conducted excluding cases with positive pathogen detection in PCR and/or rapid antigen tests from the study cohort.

### Data analysis

To assess balance between study arms, baseline variables were summarized using median and interquartile range (IQR; for numeric variables) or counts and percentages (for categorical variables) and compared between arms. For JUNO, the sample size was calculated to obtain a 95% CI of the baseline proportion of patients prescribed antibiotics in the SC arm. The required sample size for a CI with half‐width of at most 0.1 is *n* = 97, and therefore the sample size for the pilot phase was approximately *n* = 194.^23^ The sample size calculation of the JUPITER RCT is provided in the Supplementary Methods.

The CI on a population proportion was calculated using the method of Agresti‐Coull.^24^ The CI on the absolute difference between the population proportion of two independent groups was calculated using the method of Agresti‐Caffo.^24^ The CI on the relative difference between the population proportion of two independent groups was calculated using the logarithm method of Katz et al.^25^ Statistical analyses were performed with the Python programming language.

## RESULTS

### Characteristics of the study subjects

Of 260 randomized patients enrolled between March 2023 and February 2024, a total of 214 were discharged from the ED/UC and included in this analysis (SC arm, *n* = 106; MMBV arm, *n* = 108; Figure 1B). Median (IQR) age was 40.0 (28.0–55.8) years, with 57.0% female. A total of 78.5% were enrolled in the ED arm and 21.5% in the UC arm. Age, sex, race, and ethnicity were similar across trial arms (Table 2).

**TABLE 2:** Study population.

Characteristic	Characteristic name	Study population (*n* = 214)	SC arm (*n* = 106)	MMBV arm (*n* = 108)
Demographic	Age (years)	40.0 (28.0–55.8)	39.0 (27.0–56.8)	40.0 (29.8–54.2)
	Age <45 years	124 (57.9)	62 (58.5)	62 (57.4)
	45 ≤ age <65 years	59 (27.6)	29 (27.4)	30 (27.8)
	Age ≥65 years	31 (14.5)	15 (14.2)	16 (14.8)
	Female	122 (57.0)	61 (57.5)	61 (56.5)
	White	112 (52.3)	52 (49.1)	60 (55.6)
	Black or African American	77 (36.0)	42 (39.6)	35 (32.4)
	Race other	18 (8.4)	10 (9.4)	8 (7.4)
	Asian	10 (4.7)	5 (4.7)	5 (4.6)
	American Indian or Alaska Native	1 (0.5)	1 (0.9)	0 (0.0)
	Hispanic or Latino	39 (18.2)	19 (17.9)	20 (18.5)
	Not Hispanic or Latino	174 (81.3)	87 (82.1)	87 (80.6)
	ED	168 (78.5)	80 (75.5)	88 (81.5)
	UCC	46 (21.5)	26 (24.5)	20 (18.5)
Current illness^a^	Cough	198 (93.0)	97 (91.5)	101 (94.4)
	Sputum production	117 (54.9)	65 (61.3)	52 (48.6)
	Dyspnea	100 (46.9)	54 (50.9)	46 (43.0)
	Chest discomfort	97 (45.5)	44 (41.5)	53 (49.5)
	Chills	149 (70.0)	80 (75.5)	69 (64.5)
	Hematology done	134 (62.9)	62 (58.5)	72 (67.3)
	CXR done	143 (67.1)	65 (61.3)	78 (72.9)
Diagnosis^b^	Viral infection^c^	80 (37.9)	45 (42.5)	35 (33.3)
	Upper respiratory tract infection^d^	50 (23.7)	27 (25.5)	23 (21.9)
	Cough	36 (17.1)	16 (15.1)	20 (19.0)
	Fever	18 (8.5)	6 (5.7)	12 (11.4)
	Pneumonia	15 (7.1)	7 (6.6)	8 (7.6)
BV result^e^	BV result: bacterial	40 (18.9)	22 (21.0)	18 (16.8)
	BV result: equivocal	28 (13.2)	14 (13.3)	14 (13.1)
	BV result: viral	144 (67.9)	69 (65.7)	75 (70.1)

*Note*: Data are reported as median (IQR) or *n* (%).

Abbreviations: CXR, chest X‐ray; MMBV, MeMed BV; SC, standard care.

^a^ There is information missing for one patient.

^b^ Diagnosis as recorded in the medical record. Patients can be included in more than one diagnosis; showing diagnosis with *n* ≥ 15 patients; “other” not shown. Three patients were missing a diagnosis.

^c^ Viral infection diagnosis includes viral infection, viral syndrome, viral illness, viral respiratory infection, acute viral syndrome, COVID‐19, Influenza.

^d^ Upper respiratory tract infection diagnosis includes URTI, viral upper respiratory tract infection, acute maxillary sinusitis, pharyngitis, strep pharyngitis, viral pharyngitis, acute tonsillitis, acute nonrecurrent frontal sinusitis.

^e^ Two patients were missing MMBV results (one in each arm).

Predominant presenting symptoms were cough (93.0%), chills (70.0%), and sputum production (54.9%). Medical records indicated more than one discharge diagnosis for many patients (36.0%) and included viral infection (37.9%), upper respiratory tract infection (23.7%), cough (17.1%), fever (8.5%), and pneumonia (7.1%). Complete blood count (CBC) and chest X‐ray (CXR) were performed for 62.9% and 67.1% of patients, respectively. Both CBCs (58.5% vs. 67.3%) and CXRs (61.3% vs. 72.9%) were less frequently performed in the SC arm versus MMBV arm.

### Main results

The antibiotic prescription rate was 30% (95% CI 22% to 40%) in the SC arm and 24% (95% CI 17% to 33%) in the MMBV arm. This represents an absolute difference of −6% (95% CI −18% to 6%) and a relative difference of −20% (95% CI −49% to 24%).

**FIGURE 2:**
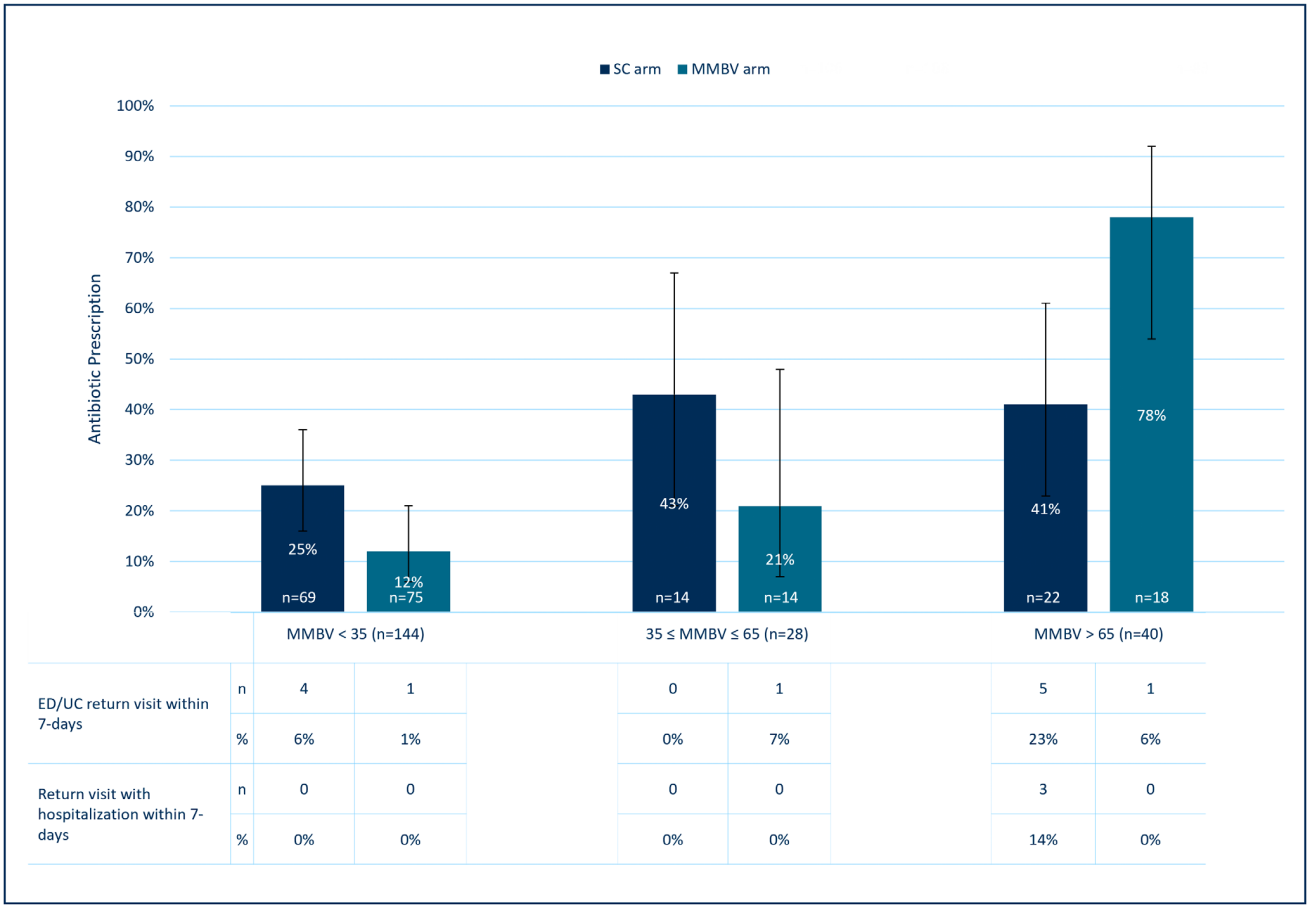
Impact of MMBV on antibiotic prescription and ED/UC return visit. Impact of MMBV on antibiotic prescription and on return ED/UC visit with or without hospitalization. The distribution of prescription rates and ED/UC return visits across MMBV scores: Patients with unnecessary antibiotic prescription (MMBV <35; left‐hand columns) and in patients with bacterial scores (MMBV >65; right‐hand columns). Two randomized patients missing MMBV scores (one in each arm) are omitted. MMBV, MeMed BV; SC, standard care; UC, urgent care center.

In an exploratory analysis, the distribution of prescription rate changes across MMBV scores was examined (Figure 2). Unnecessary antibiotic prescriptions (score < 35; *n* = 144; left‐hand columns) were given more often in the SC patients (25%, *n* = 69 [95% CI 16% to 36%] vs. 12%, *n* = 75 [95% CI 6% to 21%], respectively); an absolute difference of −13% (95% CI −25% to 0%) and a relative difference of −51% (95% CI −77% to 2%). Higher prescription rates were observed in the population with a score of >65 (*n* = 40; right‐hand columns) in the MMBV arm (78%, *n* = 18 [95% CI 54%–92%]) than the SC arm (41%, *n* = 22 [95% CI 23% to 61%]), an absolute difference of 37% (95% CI 6% to 61%) and a relative difference of 90% (95% CI 9% to 233%).

No increase in ED/UC return visit within 7 days with or without hospitalization was observed between arms overall and across the viral and bacterial MMBV subgroups (Figure 2). Overall, nine of 106 patients (8%, 95% CI 4% to 16%) had an ED/UC return visit in the SC arm, whereas this occurred for three of 107 patients (3%, 95% CI 1% to 8%) in the MMBV arm, absolute difference: ‐6% (95% CI ‐12% to 1%). In the subgroup of patients with viral MMBV scores, 7‐day ED/UC return rates were 6% (95% CI 2% to 14%) versus 1% (95% CI 0% to 8%) in SC and MMBV arms, respectively. Notably, among the patients with MMBV scores of >65, five versus one had a return ED/UC visit in the SC versus MMBV arms, of whom three of five versus zero of one were ultimately hospitalized (Figure 2), including a patient with a working sepsis diagnosis.

### Additional analysis

There were 152/214 (71%) with complete telephonic follow‐up data, 78/106 (74%) in the SC arm and 74/108 (69%) in the MMBV arm. When assuming those without telephonic data returned to the ED/UC, 35/106 (33%, 95% CI 25% to 42%) patients had a return visit to the ED/UC in the SC arm, and 36/108 (33%, 95% CI 25% to 43%) patients had a return visit to the ED/UC in the MMBV arm (Figure S1B). When removing those without telephonic data, findings similar to those of the main analysis were observed (Figure S1C). Additionally, excluding cases with positive pathogen detection in PCR and/or rapid antigen tests (*n* = 81) from the study cohort resulted in similar findings to the main analysis (Figure S1D).

## DISCUSSION

The JUNO pilot RCT demonstrated that the design and workflow of the JUPITER RCT results in a 30% antibiotic prescription rate in the SC arm. Additionally, exploratory analyses indicated that, on the one hand, MMBV availability reduced the rate of antibiotic prescriptions to patients with viral MMBV scores without increasing the rate of return ED/UC visits. On the other hand, MMBV availability increased the rate of antibiotic prescriptions to patients with bacterial scores and there were fewer hospitalizations. These findings support proceeding with the JUPITER RCT. More generally, JUNO supports MMBV's utility in aiding judicious antibiotic use when managing adult patients presenting with LRTI symptoms.

JUNO revealed a lower than anticipated prescription rate in the SC arm. This cohort reflects a real‐world population. Nevertheless, it complicates isolating the clinical question of safe antibiotic reduction, as some patients had bacterial MMBV results that led to an increase in antibiotic use to treat potentially missed bacterial infections. To address this, JUPITER's workflow will be revised so that randomization occurs after clinicians have reviewed all other diagnostic data and made prescribing and discharge decisions. Furthermore, the recently FDA‐cleared whole‐blood version of MMBV will replace the serum‐compatible version, enabling faster turnaround times and better integration into the patient journey without added burden.

JUNO was also employed to assess JUPITER's outcomes, namely, MMBV's impact on antibiotic prescribing and ED/UC return rates. Notably, JUNO supports that MMBV optimizes prescribing. On the one hand, a viral MMBV result aids in reducing unnecessary antibiotics. On the other hand, bacterial MMBV results for patients for whom the clinician was undecided can help identify patients who may benefit from antibiotics. This latter utility of MMBV is exemplified by the three patients in the SC arm who received bacterial MMBV results, but the clinician was blinded to the result and so did not prescribe antibiotics. All three ended up hospitalized within 7 days and were treated with antibiotics; one was hospitalized with a working diagnosis of sepsis. Importantly, the two desirable, but opposing, influences of MMBV on antibiotic prescribing can lead to a small net effect in real‐world settings. This net effect is dependent on patient populations (e.g., severity of illness) and antibiotic prescribing behavior of the individual clinician and the setting.

This optimization effect on antibiotic prescription was demonstrated recently in a pragmatic interventional study conducted at a UC network, where MMBV was ordered for 3920 adults.^26^ Clinicians altered their prescribing behavior based on MMBV, reducing prescriptions for patients with viral MMBV results and increasing prescriptions for patients with bacterial MMBV results. Test adherence was associated with fewer hospitalizations in 7‐day follow‐up.

JUNO focused specifically on patients presenting with symptoms of LRTI. As expected, only few had confirmed pneumonia as their discharge diagnosis.^27^, ^28^ This patient population was chosen since it is well documented that it is difficult to differentiate bacterial from viral infections in this population.^29^ The inclusion criteria were fever (either tactile or measured) and cough, sputum production, dyspnea, or auscultation abnormality rather than a confirmed LRTI. These inclusion criteria capture the heterogeneity of patients presenting to the ED/UC, representing how the test would be used in real‐world settings at the timepoint of diagnostic uncertainty.

Higher rates of CBC and CXR testing were observed in the MMBV arm. CBC was ordered before randomization and therefore testing rates could not have been influenced by the MMBV result. The timing of the CXR requisition and its formal result was not documented. Of note, in JUPITER's revised workflow, randomization will occur after CBC and CXR results are available, although there will not be any restriction on ordering additional testing after randomization should the clinician deem it warranted.

We excluded patients who were admitted to the hospital. This approach stemmed from the understanding that the clinical dilemma of the ED/UC clinician is different for patients they decide to admit. Previous studies have shown that antibiotic overuse is more prevalent upon discharge.^30^, ^31^ We suspect that for admitted patients, the clinician sometimes defers the decision whether to prescribe antibiotics to the hospitalist, who they know will have access to additional test results (e.g., cultures) and reevaluations. We think the present study design ensures that findings are applicable to the ED/UC community. Further studies are warranted to evaluate the impact of the test on appropriate antibiotic use for admitted patients. Such studies should include assessment of antibiotic treatment days and antibiotic cessation as well as antibiotic initiation and ensure that the hospitalists also receive training on interpreting the test.

Cost and reimbursement frameworks can be significant drivers or barriers to test adoption; these are different in the ED versus UC setting. Several health economic models support that MMBV is cost‐effective.^32^, ^33^ Although anecdotal, the missed bacterial infections in the SC arm who returned to hospital would incur considerable costs and point to a potential cost benefit of MMBV testing. In JUPITER, relevant data will be collected to examine the health economic effect of introducing MMBV.

## STRENGTHS AND LIMITATIONS

A strength of JUNO is that it served as a pilot to examine if JUPITER's design indeed addresses the question: Does MMBV reduce unnecessary prescription without associated adverse outcomes? Additional strengths are inclusion of multiple sites, clinicians, and settings to support generalizability of the findings. In this trial design, the clinicians were given antibiotic prescribing recommendations based on MMBV, but there was not an embedded antibiotic stewardship program requiring adherence to the result. This pragmatic approach is a strength as it reflects real‐world use of diagnostic tests as an adjunct to clinical judgment alongside other tests. This pragmatic approach could also be considered a limitation, as it may underestimate the full potential of the test's impact compared to workflows that enforce adherence, as performed previously for procalcitonin.^5^, ^6^ An additional limitation is the possibility that some patients not answering the phone sought additional medical care but went to a different facility. We present two analyses to understand the potential impact of patients without telephonic follow‐up data: one where it is assumed all patients not answering the phone returned to an ED/UC and a second focused on the subgroup of patients with telephonic data. Another limitation is that data on length of stay in the ED/UC were not collected in JUNO; these parameters will be recorded in JUPITER. Additionally, a limitation is that patient enrollment occurs only during the research team's working hours. Lastly, a limitation is that the determination that antibiotics are unnecessary is based on the MMBV viral result and is not corroborated by an adjudication‐based reference standard infection etiology. The high diagnostic accuracy of MMBV compared to a rigorous adjudication‐based standard demonstrated in multiple previous studies underpins this assumption.^10^, ^11^, ^12^, ^13^, ^14^, ^15^, ^16^, ^17^, ^18^, ^19^

One could argue that given the high availability of rapid pathogen testing (PCR and rapid antigen tests), clinicians may order fewer blood tests for patients with a positive pathogen detection at the ED/UC. Notably, a serial testing workflow does not capture the potential for patients to have a bacterial‐viral coinfection.^10^, ^12^, ^34^ Furthermore, a recent systematic review and meta‐analysis of RCTs showed that rapid viral testing was not associated with reduced antibiotic use in the ED.^9^ Here, we observed similar reductions in unnecessary antibiotic prescription across the study population also after excluding cases with a positive detection. This finding supports the MMBV's utility regardless of pathogen testing.

## CONCLUSIONS

In conclusion, JUNO indicated how to improve the design of the JUPITER randomized controlled trial. Additionally, we observed that MeMed BV reduced unnecessary antibiotic prescriptions in patients with viral MeMed BV scores presenting to the ED/urgent care center with symptoms of lower respiratory tract infection without increasing rates of ED/urgent care center return visits within 7 days. Conversely, we observed that MeMed BV increased antibiotic prescriptions in patients with bacterial scores and there were fewer hospitalizations. The JUPITER randomized controlled trial and additional real‐world evidence studies are warranted to corroborate our findings.

## AUTHOR CONTRIBUTIONS

Adam J. Singer, Judd E. Hollander, Efrat R. Kean, Hope Ring, Sergey Motov, Joby Thoppil, Phyllis Hendry, Salim Halabi, Andrew C. Meltzer, Gary F. Headden, Tal Brosh‐Nissimov, David Zeltser, and Chad M. Cannon contributed to design the trial, execution, analysis of the data, and draft and review the manuscript. W. Frank Peacock and Karina M. Soto‐Ruiz supervised the conduct of the trial and data collection, managed the data (including quality control), and reviewed the manuscript. All authors contributed substantially to its revision. Adam J. Singer takes responsibility for the paper as a whole.

## FUNDING INFORMATION

This project has been supported in part with federal funds from the Department of Health and Human Services; Administration for Strategic Preparedness and Response; Biomedical Advanced Research and Development Authority (BARDA), under contract number 75A50123C00041. In addition, MeMed is a primary sponsor of this study and DiaSorin Inc. is also a sponsor.

## CONFLICT OF INTEREST STATEMENT

AJS—research grants from AstraZeneca, Brainbox, Spectral; consultant for AstraZeneca. CMC—research grants from Abbott, AstraZeneca, Brainbox, Roche, and MeMed. WFP—research grants from Abbott, Brainbox, CSL‐Vifor, Quidel, Roche, and Siemens; consultant for Abbott, Astra‐Zeneca, Biocogniv, Brainbox, Bristol Meyers Squibb, Janssen, Osler, Quidel, Roche, Siemens, Spinchip, and Werfen; stock/ownership interests in AseptiScope Inc., Brainbox Inc., Biocogniv, Inc., Braincheck Inc., Coagulo Inc., Comprehensive Research Associates LLC, Comprehensive Research Management Inc., Emergencies in Medicine LLC, Lucia Inc., Prevencio Inc., RCE Technologies, ROMTech, ScPharma, Trivirum Inc., and Upstream Inc. ACM—research grants from Abbott, AstraZeneca, Biomerieux, CDC, Hologic, MeMed, Vapotherm, and 1 Eq Inc.; consultant for Vapotherm and Biomerieux. TBN—received honoraria from Gilead, GSK, AstraZeneca, MSD, and Medison. The other authors declare no conflicts of interest.

## Supporting information


Data S1.

## Data Availability

The data that support the findings of this study are available on request from the corresponding author. The data are not publicly available due to privacy or ethical restrictions.
