# Supplementary material for: Effect of host‐protein test (TRAIL/IP‐10/CRP) on antibiotic prescription and emergency department or urgent care center return visits: The JUNO pilot randomized controlled trial
Source: Acad Emerg Med. 2025 Apr 18;32(9):975–84. doi: 10.1111/acem.70031 (PMC12435126; doi:10.1111/acem.70031)
Supplement: Supplementary file 1 — Data S1. [file ACEM-32-975-s001.docx]

**Supplementary Materials**

JUNO is named after the NASA mission to Jupiter that started on August 5, 2011.

**Supplementary Methods**

JUPITER’s Outcomes

The statistical framework for safe antibiotic reduction is based on previous studies.^1–4^

Acceptance criteria:

- For the primary outcome, the null hypothesis was rejected if the upper limit of the two-sided 95% CI on the absolute difference in antibiotic prescribing rate was <0. (superiority).
- For the secondary outcome, the null hypothesis was rejected if the upper limit of the two-sided 95% CI on the absolute difference in return visit rate was below the non-inferiority margin of 7.5%.

The frequency of the secondary outcome was based on data from two trials with similar design and scope.^1,2^ Considering the population that is sufficiently ill to present at the ED/UC, but for whom discharge is considered, the overall risk of return ED/UC visits within 7-days was estimated as 10%. To define non-inferiority regarding the secondary outcome, a 7.5% absolute difference was chosen as a clinically acceptable limit.^1–4^

JUPITER’s Sample Size

*For the primary outcome:*

The proportion of patients prescribed antibiotics in the MMBV arm (p2) will be compared to the proportion in the SC arm (p1). The hypotheses that will be tested are:

*H_0_: p_2_ ≥ p_1_*

*H_1_: p_2_ < p_1_*

The endpoint will be met if the null hypothesis is rejected with probability of type I error (alpha) of 0.025.

Detailed power analysis was conducted to ensure attainment of this endpoint, assuming that the expected proportion of patients prescribed antibiotics is 85% in the BV arm and 95% in the control arm. The smallest number of patients per arm (in multiples of 50) providing power of 90% is 200 in each arm, therefore at least 400 patients need to be included.

We estimate that 15% of enrolled patients will be excluded from the study, therefore the total number of enrolled patients required is 471.

*For the secondary outcome:*

Originally, the trial secondary outcome included: death, vasopressor use for >1h, mechanical ventilation, diagnosis of lung abscess/empyema occurring within 28 days after enrollment. However, as the prevalence of these outcomes was below 1% for all populations, we decided to add a clinical outcome of return ED/UC visits within 7-days as a more clinically relevant outcome for this mild population. The change is documented in protocol amendment and approved by IRB.

The proportion of patients reaching the clinical outcome of return ED/UC visits within 7-days in the MMBV arm (p2) will be compared to the proportion in the SC arm (p1). The hypotheses that will be tested are:

*H_0_: p_2_ - p_1_ ≥ 0.075*

*H_1_: p_2_ - p_1_ < 0.075*

The endpoint will be met if the null hypothesis is rejected with probability of type I error (alpha) of 0.025.

Detailed power analysis was conducted to ensure attainment of this endpoint, assuming that the expected proportion of patients experiencing an adverse clinical outcome is 10% in both arms. The smallest number of patients per arm (in multiples of 10) providing power of 85% is 310 in each arm, therefore at least 620 patients need to be included.

We estimate that 15% of enrolled patients will be excluded from the study, therefore the total number of enrolled patients required is 730.

*Overall sample size:*

Determination of sample size was dependent on the hypothesis test for the secondary outcome, as this required a larger sample size than the primary outcome. The total number of enrolled patients required is therefore 730.

**Supplementary Figures**

Figure S1: Impact of MMBV on antibiotic prescription and ED/UC return visit across different cohorts


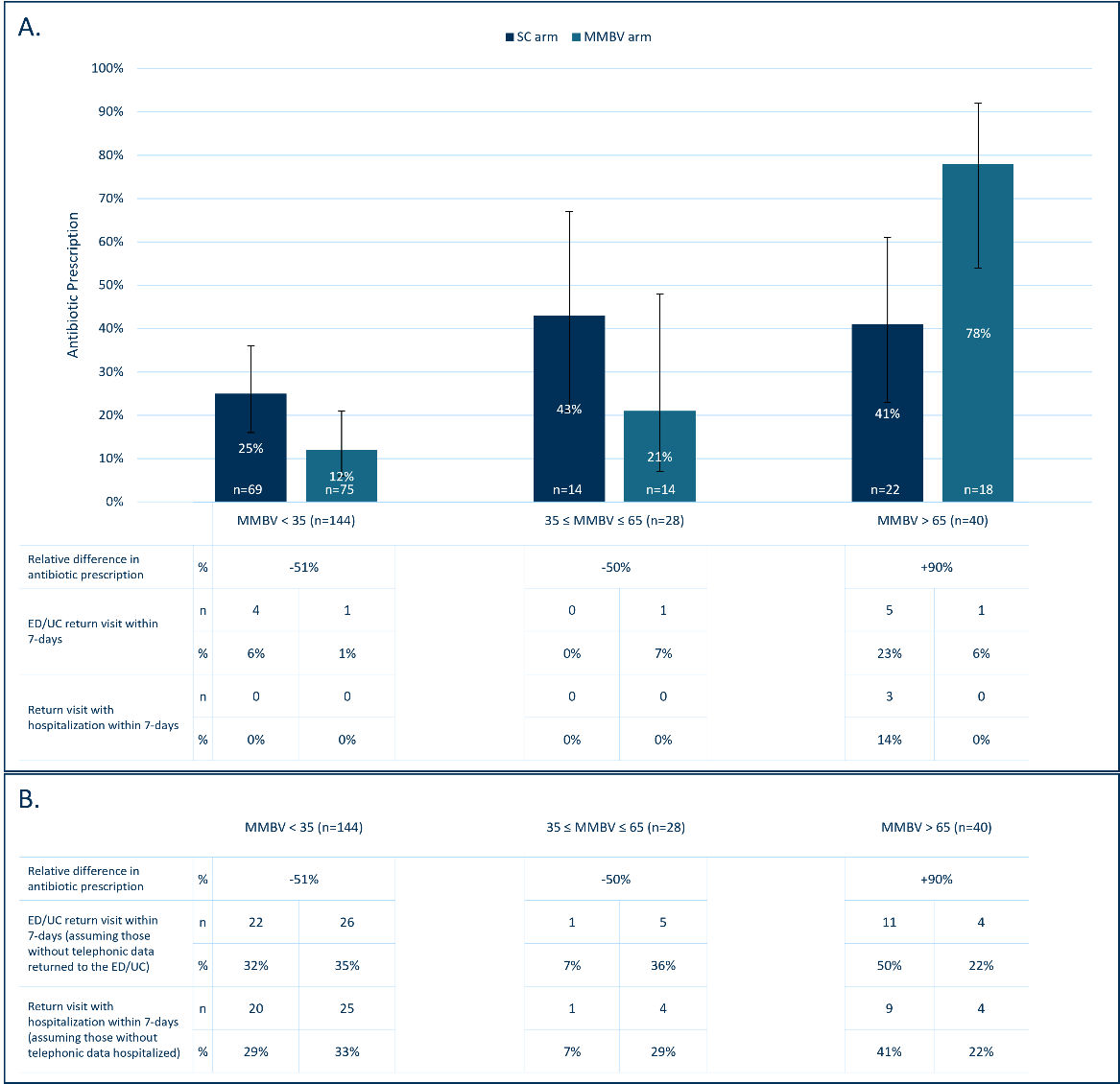


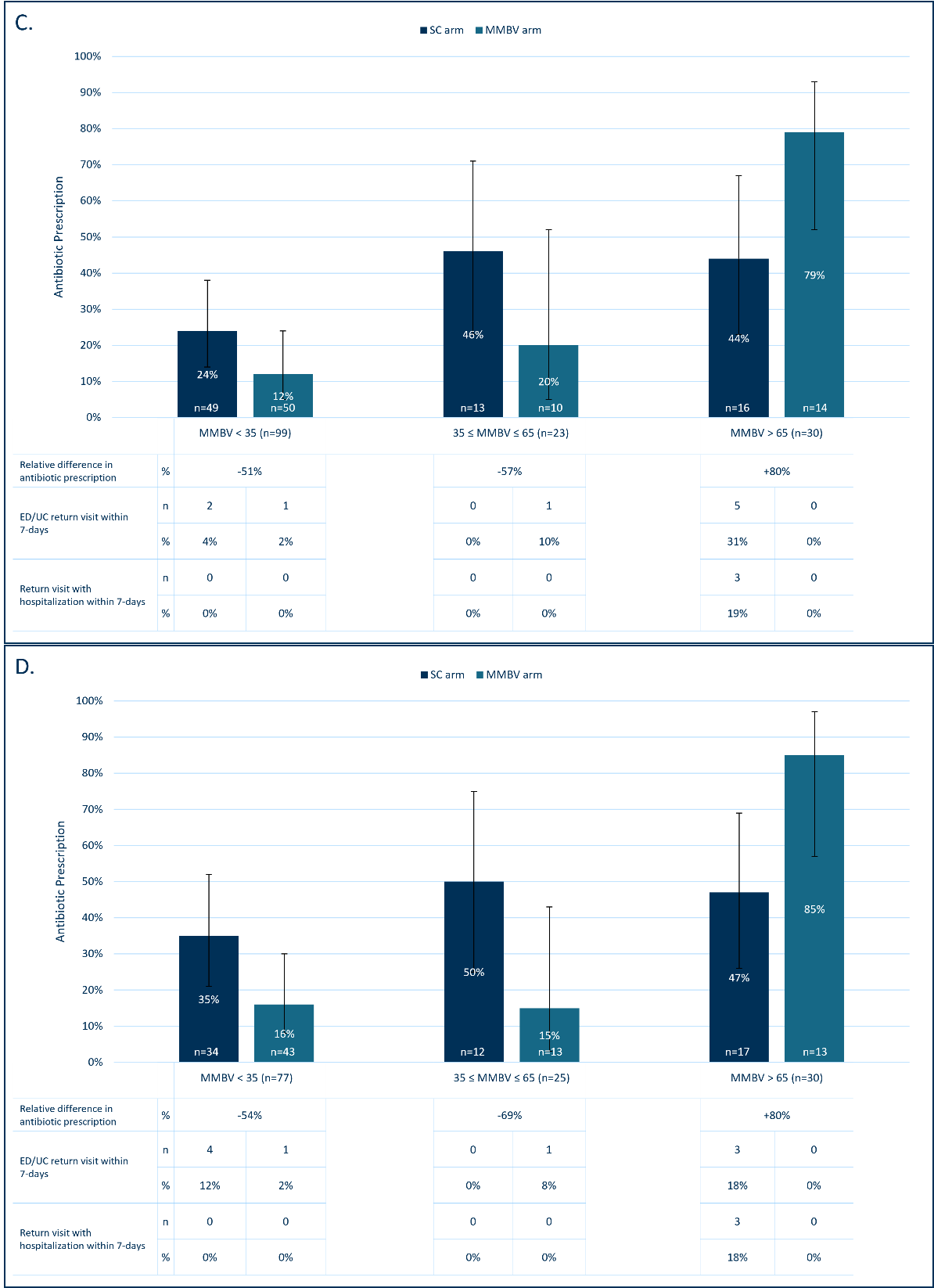


Figure S1 legend: Impact of MMBV on antibiotic prescription and ED/UC return visit +/- hospitalization across different cohorts and across MMBV scores.

(A) Entire cohort (B) Entire cohort assuming those without telephonic data returned to the ED/UC. (C) Sub-cohort with complete telephonic data (D) Sub-cohort without positive pathogen detection in PCR or rapid antigen tests.

**Supplementary References**

1. Schuetz P, Christ-Crain M, Thomann R, et al. Effect of Procalcitonin-Based Guidelines vs Standard Guidelines on Antibiotic Use in Lower Respiratory Tract Infections: The ProHOSP Randomized Controlled Trial. JAMA 2009;302(10):1059–66.

2. Huang DT, Yealy DM, Filbin MR, et al. Procalcitonin-Guided Use of Antibiotics for Lower Respiratory Tract Infection. N Engl J Med 2018;379(3):236.

3. Tsalik EL, Rouphael NG, Sadikot RT, et al. Efficacy and safety of azithromycin versus placebo to treat lower respiratory tract infections associated with low procalcitonin: a randomised, placebo-controlled, double-blind, non-inferiority trial. Lancet Infect Dis 2023;23(4):484–95.

4. Does Y van der, Limper M, Jie KE, et al. Procalcitonin-guided antibiotic therapy in patients with fever in a general emergency department population: a multicentre non-inferiority randomized clinical trial (HiTEMP study). Clin Microbiol Infect 2018;24(12):1282–9.
